# Understanding biological mechanisms underlying adverse birth outcomes in developing countries: protocol for a prospective cohort (AMANHI bio–banking) study

**DOI:** 10.7189/jogh.07.021202

**Published:** 2017-12

**Authors:** AMANHI (Alliance for Maternal and Newborn Health Improvement) Bio–banking Study group), Abdullah H Baqui, Rasheda Khanam, Mohammad Sayedur Rahman, Aziz Ahmed, Hasna Hena Rahman, Mamun Ibne Moin, Salahuddin Ahmed, Fyezah Jehan, Imran Nisar, Atiya Hussain, Muhammad Ilyas, Aneeta Hotwani, Muhammad Sajid, Shahida Qureshi, Anita Zaidi, Sunil Sazawal, Said M Ali, Saikat Deb, Mohammed Hamad Juma, Usha Dhingra, Arup Dutta, Shaali Makame Ame, Caroline Hayward, Igor Rudan, Mike Zangenberg, Donna Russell, Sachiyo Yoshida, Ozren Polašek, Alexander Manu, Rajiv Bahl

**Affiliations:** 1 Center for Maternal and Newborn Health, Department of International Health, Johns Hopkins Bloomberg School of Public Health, Baltimore, Maryland, USA; 2 Department of Pediatrics and Child Health, Aga Khan University, Pakistan; 3 Centre for Public Health Kinetics, India and Public Health Laboratory–IdC, Pemba, Tanzania; 4 University of Edinburgh: Centre for Global Health Research, Old Medical School, Edinburgh, Scotland, UK; 5 University of Split School of Medicine, Split, Croatia; 6 Gen–info Ltd, Zagreb, Croatia; 7 World Health Organization (MCA/MRD), Geneva, Switzerland

## Abstract

**Objectives:**

The AMANHI study aims to seek for biomarkers as predictors of important pregnancy–related outcomes, and establish a biobank in developing countries for future research as new methods and technologies become available.

**Methods:**

AMANHI is using harmonised protocols to enrol 3000 women in early pregnancies (8–19 weeks of gestation) for population–based follow–up in pregnancy up to 42 days postpartum in Bangladesh, Pakistan and Tanzania, with collection taking place between August 2014 and June 2016. Urine pregnancy tests will be used to confirm reported or suspected pregnancies for screening ultrasound by trained sonographers to accurately date the pregnancy. Trained study field workers will collect very detailed phenotypic and epidemiological data from the pregnant woman and her family at scheduled home visits during pregnancy (enrolment, 24–28 weeks, 32–36 weeks & 38+ weeks) and postpartum (days 0–6 or 42–60). Trained phlebotomists will collect maternal and umbilical blood samples, centrifuge and obtain aliquots of serum, plasma and the buffy coat for storage. They will also measure HbA1C and collect a dried spot sample of whole blood. Maternal urine samples will also be collected and stored, alongside placenta, umbilical cord tissue and membrane samples, which will both be frozen and prepared for histology examination. Maternal and newborn stool (for microbiota) as well as paternal and newborn saliva samples (for DNA extraction) will also be collected. All samples will be stored at –80°C in the biobank in each of the three sites. These samples will be linked to numerous epidemiological and phenotypic data with unique study identification numbers.

**Importance of the study:**

AMANHI biobank proves that biobanking is feasible to implement in LMICs, but recognises that biobank creation is only the first step in addressing current global challenges.

Sub–Saharan Africa and south Asia are the sub–regions with the highest proportion of the global burden of over 289 000 maternal deaths, 6 million child deaths and 2.6 million stillbirth [1–3]. To improve survival and secure attainment of developmental potential of both mothers and their babies, holistic approaches that have future applicability are warranted. A better understanding of the biological mechanisms underlying adverse birth outcomes (such as eclampsia, intrauterine growth restriction, preterm births and stillbirths) and their relationships with various phenotypic, epidemiologic and more importantly epigenetic characteristics will provide a gateway to addressing these challenges.

Evidence from high–income settings suggests that biobanks, which are repositories of biological samples with data linked to individual subjects’ characteristics, may provide a sustained platform with infrastructure for research and discovery of biological mechanisms underlying those leading causes of deaths [4–6]. These mechanisms may have roots in endogenous and exogenous factors (eg, genetic composition, nutrition, environment, etc.) [7–9]. Biological molecules comprising glycomes, proteomes, lipidomes, and other metabolomes that circulate in the blood, other human tissues and body fluids have been linked with detection of risks of adverse maternal and fetal outcomes. For instance, soluble fms–like tyrosine kinase (sFlt–1 or sVEGFR–1) have been found to blunt the beneficial effects of proangiogenic factors on maternal endothelium with consequences such as proteinuria and hypertension (pre–eclampsia) [10–12]. Other biomarkers such as soluble endoglin (s–Eng), P–selectin, Cell free fetal DNA (cfDNA), placental protein 13 (PP–13) are being evaluated as predictors of pre–eclampsia and intrauterine growth restriction (IUGR) [13–18].

However, these factors may vary between developed and developing country populations. If these hypothesized biomarkers prove to be important predictors for these adverse outcomes in developing country settings too, they could potentially allow early risk assessment of pregnant woman in order to promote timely referral and optimal management. The current distribution of bio–repositories is skewed to high income countries (HICs) and their focus of research on ageing and chronic diseases, arguably, may have very limited immediate value for low– and middle–income countries (LMICs) [19,20]. Translating knowledge acquired from developed country settings to implement interventions in developing country settings without testing could be a risky investment. Biobanks are largely lacking in sub–Saharan Africa and South Asia.

Prior to recent initiatives such as the Human Heredity and Health in Africa (H3Africa) by the National Institute of Health, USA [21,22], only few African countries had biobanks [23,24] and almost none in south Asia. In developing country biobanks, biological samples are collected at clinics for focused research into specific infectious diseases and, coupled with weak health systems and poor access to health care, data are rarely systematically collected to make them useful in describing population dynamics, disease and death. Large–scale, population–level epidemiological research with capability to acquire biological specimen that can be linked to morbidity and present and future mortality are crucial if the advantages provided by newly–available high throughput analytical technologies are to be exploited to maximise the public health and clinical relevance of research activities [20].

There are suggestions that developing countries do not have the capacity (the legislation, human resource and logistics such as reliable power supply) to establish and maintain biobanks [25]. Whilst this may be true for now, context also plays an important role in diseases and deaths and so capacity building in these settings is paramount. Fortunately, with the ever–increasing availability of and great advances in high throughput technologies at progressively decreasing cost [20], such biological specimen assays to identify biological markers that predict or are associated with pregnancy–related outcomes, growth and development will have direct global relevance especially for LMICs.

The Alliance for Maternal and Newborn Health Improvement (AMANHI) [26,27] initiative aims to establish the best–characterized cohort of pregnant women and their babies in sub–Saharan Africa and south Asia. This study will contribute to advancing knowledge on key pregnancy and birth outcomes on a sustained research platform, prove the principle that such initiatives are feasible to implement in LMICs and develop local capacity around biobanking and the use to explore future hypotheses.

## Methods

### Study design

This is a population–based, prospective cohort study to collect detailed epidemiological and biological data.

#### Objectives

1. To uncover biological markers as predictors of important maternal and foetal outcomes. To do this AMANHI biorepository study will:
  - Conduct case–control studies to identify biomarkers that can predict (pre–) eclampsia, preterm births, IUGR, and stillbirths.
  - Replicate the role of genetic variants which have been identified in high–income settings as important determinants of these outcomes through genome–wide association and candidate gene approaches e.g. PSG11, INHBB, ACVR2A, etc. (pre–eclampsia) [11,17,18] and ADCY5, CDKAL1, HHEX–IDE, GCK, etc. (preterm births/low birthweight) [13,28,29].
  - Evaluate the validity of the most commonly proposed existing biochemical, nutritional and inflammatory biomarkers (in serum, plasma or urine), which have been identified from hospital–based studies in high income countries (such as PAPP–A, AFP and inhibin–A (INH), homocysteine, sFlt–1, etc.) [14,30]
2. To facilitate future discoveries in maternal, foetal and neonatal health as new and more feasible methods become available, by establishing a bank/repository of biological samples which collects, stores and maintains samples in a harmonized way across sites in developing countries.

### Overview of the AMANHI Biobanking Study protocol

An on–going surveillance system with longitudinal follow–up of a dynamic cohort of women of reproductive age has been established in all sites–Bangladesh (Sylhet), Pakistan (Karachi) and Tanzania (Pemba Island)–in South Asia and sub–Saharan Africa. These sites were selected because they represent predominantly rural populations within geographic regions of the world with the highest maternal and foetal mortality burden and centres have proven excellent track–record of accomplishment of international research, good leadership and on–going trials will not interfere with the AMANHI biobanking study protocols. All resident women with early pregnancies (before 20 gestational weeks), who intend to stay in the study areas for the entire duration of follow–up, are consented for collection of epidemiological data as well as biological samples for the study. The overall sampling frame is 3000 trios (1000 per site), and the collection process is expected to be functional from August 2014 to June 2016. Unpublished results from the parent studies show less than 5% attrition rates in the population. Many study procedures are similar across sites but the following are site–specific settings for the study:

In Bangladesh this study is being implemented within the Bangladesh Maternal Infection and Preterm Birth (MIST) study by the Project for Advancing the Health of Newborns and Mothers (PROJAHNMO) and the John Hopkins University. All pregnant women are identified through monthly pregnancy surveillance by community health workers (CHWs) and village health workers (VHWs). All pregnancies are confirmed via a strip–based pregnancy tests (Diaspot, marketed by BRESTA) administered by CHW. CHWs, supported by senior staff and study physicians (where needed) consent and enrol pregnant women for the study. All enrolled mothers are placed under e–surveillance using cell phone. Contact numbers are exchanged between women and respective CHWs for ease of communication. The study area is served by six health facilities operated by the Bangladesh Ministry of Health and Family Welfare (MOHFW). Four of them are first level health facilities staffed by a physician or paramedic; these facilities provide a range of preventive care services including antenatal care and normal delivery care. The remaining two are 31 bed hospitals with doctors, nurses, in–patient care and basic laboratory facilities.

In Pakistan, the Biobanking study will be conducted in two peri– urban communities of Karachi, Ibrahim Hyderi (IH) Goth and Bilal Colony Rehri (RG) Goth within the pregnancy and newborn surveillance as part of the Aetiology of Newborn Infections Study in Asia (ANISA) study. Trained CHWs conduct 3–monthly surveillance to identify pregnancies and conduct confirmatory urine dipstick test. Women with confirmed pregnancies are recruited for an ultrasound scan in a study facility whereupon they are enrolled if found eligible. CHWs will be supported by research assistants and study investigators will follow–up on all enrolled women to recruit them to the study centres for sample collection. Phenotypic and epidemiological data will be collected at the homes of the participants. The study area is served by several health facilities–primary to tertiary–but sample collection will be done at IH health centre and the Aga Khan University Hospital.

In Tanzania, the study is being carried out in two of the four districts or “Shehias” of Pemba, the smaller of the two islands of the Zanzibar archipelago. It is a collaboration between the from Johns Hopkins University and local Investigators from Pemba Health Laboratory–IDC and Ministry of Health, Zanzibar. There is an ongoing delivery and neonatal surveillance at facilities and within communities throughout the island as part of the ongoing chlorhexidine (CHx) application to the umbilical cord trial. Consequently, close contacts and communication has been established between the study team, all the Maternal and Child Health (MCH) staff, TBAs and all facilities where deliveries occur on the island. There is a 2–monthly surveillance for identifying pregnancies by CHWs. CHWs provide menstrual calendars to women to record menstrual periods. If the period is missed at two consecutive months, pregnancy tests are performed by local MCH staff or TBA together with a study supervisor. Women are consented for screening ultrasound at study centres and enrolled if found to be eligible. An elaborate system of tracking of pregnant women with the aid of geographical information systems and exchange of mobile contact numbers between participants’ families and study staff. Immediate information about all deliveries is communicated to a central study call centre and the informant is directly linked to the appropriate study team member and designated MCH staff is arranged. This call also helps to get all information regarding stillbirths and location/directions to the household for home deliveries. Mobile clinics are used for following up enrolled women. Two main facilities in the district are being used for biological sample collection.

**Figure 1:**
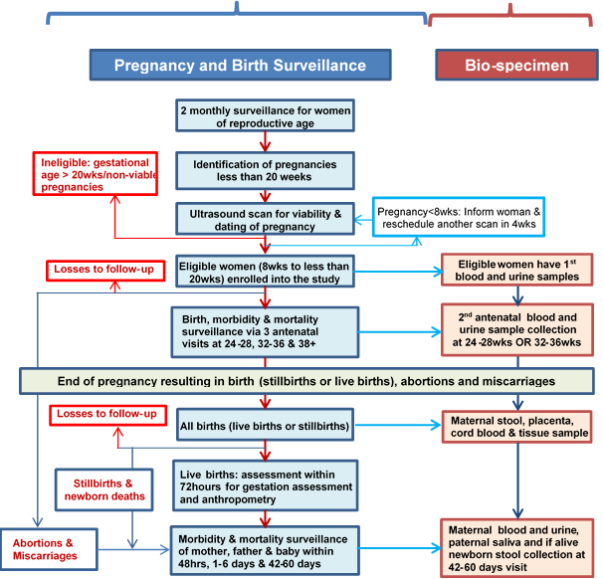
Overview of the study protocol for surveillance and bio–specimen collection within the AMANHI bio–repository study.

**Figure 1** shows an algorithm of the strategy for implementing the AMANHI study. The key components of the study protocol are pregnancy and birth surveillance, ultrasound scan to date pregnancy and to enrol women, epidemiological and phenotypic data collection, and biological sample collection, processing and storage.

*Pregnancy and birth surveillance*: Site specific protocols are as described above. Essentially, in all the three AMANHI sites, all households have been provided unique identification (ID) numbers and have had their geographical coordinates collected and linked to a database. Trained fieldworkers (FWs), predominantly women, perform home visits every 2–3 months to all women of reproductive age in the study area to enquire about pregnancy. If a woman reports or suspects a pregnancy, FWs ascertain the gestational age using the date of her last menstrual bleeding and conduct a urine pregnancy test to confirm. Pregnant women who provide consent undergo a screening ultrasound to date the pregnancy more precisely.

*Consenting*: Fieldworkers consent pregnant women, in their local or preferred languages, to undergo a screening ultrasound scan to date pregnancies accurately. They enroll women if the ultrasound–estimated gestational age of the pregnancy is within the eligibility cut–offs of 8 to 19 weeks. Women are consented for the screening scan, follow–up and biosample collection. However, at each sample collection contact, women are required to provide consent for their babies and their own sample taking. Women’s husbands (fathers of the babies) also consent for their saliva sample collection.

*Early pregnancy dating ultrasound (gold standard for gestational age) and enrolment*: This is the entry point into the study. Recruited women undergo this dating scan at designated AMANHI facilities to determine foetal viability and measure foetal biometric parameters for gestational age determination. Women whose pregnancies are found to be between 8 and 19 weeks of gestation are considered eligible for enrolment. Harmonized protocols have been developed by a consultant in obstetrics and gynaecology and all study sonographers (medically–trained professionals with additional specialist training in ultrasonography) were trained in strategies to improve image quality and to obtain accurate measurements of the biometric parameters. Crown–rump length (CRL) is the biometric measure of choice for foetuses less than 14 weeks whereas bi–parietal diameter (BPD) and femur length (FL) are taken for foetuses at 14 weeks of gestation or more. Quality assurance measures include expert review of images internally (within site) for a random 10% of study subjects and externally (centrally by the consultant in obstetrics and gynaecology) for a random 5% of subjects. Each image is scored based on a quality checklist and sonographers are provided routine feedback for quality improvement.

*Late Pregnancy Ultrasound:* On a subset of mothers, we are also conducting late pregnancy biometry and testing the accuracy of transcerebellar diameter measurements in late pregnancy for gestational age assessment, as compared with early ultrasound based assessment. In the late pregnancy scans, the biometric measurements include bi–parietal diameter (BPD), head circumference (HC), femoral length (FL), abdominal circumference (AC), and transcerebellar diameter (TCD). For this sub–study, all images are being reviewed with stringent quality control and scoring, and routine feedback provided to sonographers.

### Phenotypic and other epidemiological data collection

Trained study fieldworkers (FWs) conduct four home visits to the enrolled women; at baseline (immediately after enrolment), and at 24–28 weeks, 32–36 weeks and after 37 completed weeks of pregnancy to collect routine study data as detailed in **Table 1**. After delivery, three additional visits are made, within 72 hours, on the fourth to the seventh day after birth and after 42 days [26]. Women or newborns with a morbidity or an abnormal urine test and BP results are referred for appropriate care within health facilities. Core variable tables containing the minimum data that should be collected across all sites have been developed. They ensure uniform data are collected across all sites and specify the type of question, the response options and how the data are presented in the databases (text, numeric or string). These core variables tables were translated into questionnaires for phenotypic and epidemiological data collection across sites.

**Table 1:** Type of epidemiological and phenotypic data being collected during home visits

Modality	Details being collected in AMANHI biorepository study	Time of collection
Background characteristics	Demographic, socio–economic, other characteristics of the woman and her household and an asset inventory to be used in constructing an asset index for classifying women into wealth quintiles	Baseline visit
Medical history	Previous obstetric and gynaecological history, history of birth defects and congenital anomalies among previous babies, stillbirths and IUGRs; previous medical and surgical history including medicinal prescription drugs taken or being taken for chronic diseases and periodontal diseases	Baseline and antenatal (AN) visits
Risk factors and exposures	Cigarette smoking, alcohol ingestion, smoke from biomass cooking fuels, occupational chemical exposures, strenuous physical work and the use of narcotics and other drugs	Baseline visit
Depression screening	Depression screening using the 9–question patient health questionnaire (PHQ–9)	AN and postnatal (PN) visits
Anthropometry	Maternal weight and height, maternal mid–upper arm circumference and abdominal girth	Baseline, AN and PN visits
Reported morbidity	Questions about any illness or complications during pregnancy, childbirth and the postpartum period. Care–seeking, hospitalizations and treatment received for any morbidity	AN and PN visits
Assessment for pre–eclampsia	Measurement of blood pressure using a digital (Microlife®) sphygmomanometer (calibrated for hyperdynamic circulation in pregnancy) and testing urine for proteins (Uristix®)	All visits except delivery visits
Nutritional assessment	Food frequency questionnaire	One AN visit and Post day 42 PN visit

There is a passive surveillance system in health care facilities where trained hospital FWs collect clinical data on any facility attendance by women enrolled in AMANHI. These data cover the reason for attending the facility, the details of the treatment given and the outcomes.

Harmonized protocols have been used across sites for training study supervisors (who are experienced fieldworkers who have had experience working in health facilities and provided additional training for newborn handling and assessment) to conduct neuromuscular, physical and feeding maturity assessment within 72 hours of the birth on all babies born to women enrolled in the AMANHI study. They also take neonatal anthropometric measurements (head, chest and mid–upper arm circumference, foot length and breast bud diameter) on the baby during these visits. Trained study clinicians validate 5%–15% assessments through repeat assessments also within the 72–hour window period after birth.

After 42 days of the birth, fieldworkers visit families to collect epidemiological and phenotypic data and whereupon women exit the study.

In case of a maternal, foetal (stillbirth) or neonatal death, uniform protocols and training have been provided to supervisors in all sites to conduct home visits to conduct verbal autopsy interviews with reliable informants to elicit the circumstances leading to the death and any relevant information that may help identify the medical cause of death. Harmonized methods (based on principles of the International Classification of Diseases) are being used to assign the causes of deaths and complete death certificates for each death on a specially designed software platform [27].

### Biospecimen collection and processing

A sampling scheme with a sequence of time points to obtain maternal blood and urine, maternal stool, umbilical cord blood and tissue, placenta tissue and membranes, newborn stool and saliva samples (where cord blood is not available), and paternal saliva samples are used. Samples are collected at enrolment, at either 24–28 weeks or 32–36 weeks antenatal visit, at delivery and after 42 days of the delivery. Participants are randomised for antenatal maternal blood and urine collection at either 24–28 weeks or 32–36 weeks gestation in a ratio 2:1. **Table 2** shows the samples collected, the timing of collection and the main extraction from these samples in AMANHI.

**Table 2:** Timing of collection, processing and/or main extraction for AMANHI biological samples

Sample type	Timing of collection	Processing & planned use of the sample
Maternal blood	Enrolment, 24–28 weeks or 32–36 weeks, postnatal day 42–60	For DNA extraction, HbA1C analysis, and serum/plasma extraction, aliquoting and storage
Maternal urine	Enrolment, 24–28 weeks or 32–36 weeks, postnatal day 42–60	Uncentrifuged and centrifuged sample, biochemical and pathological analysis
Cord blood sample	At birth	DNA extraction, HbA1C analysis, and serum/plasma extraction, aliquoting and storage
Cord tissue samples	At birth	RNALater, alcohol, flash frozen and formalin sample
Placenta tissue samples	At birth	RNALater, alcohol, flash frozen and formalin sample
Placenta membrane samples	At birth	RNALater, alcohol, flash frozen and formalin sample
Maternal faeces	At birth	Maternal faecal microbiome
Paternal saliva	Antenatal or postnatal	Paternal DNA
Fetal faeces	Postnatal day 42–60	Newborn faecal microbiome

Standardized protocols are being implemented across all sites. Blood samples of the mother and from the umbilical cord are collected into pre–labelled tubes, centrifuged and serum, plasma and buffy coat aliquots obtained. Aliquots of whole blood samples are also used to perform HbA1C assay and spots were also placed in Whatman cards and dried. Maternal urine samples are similarly centrifuged and RNALater is mixed with sediments and aliquots taken for storage.

Placenta samples are harvested and processed within 30 minutes of delivery and photographs of the surfaces taken. Full thickness tissues samples are harvested in four areas, three of which have a thin layer of maternal tissues sliced off the surface. Samples of the membranes and umbilical cord are also taken. Placental tissue samples are stored in RNALater, alcohol, or are flash frozen. A sample is also stored in formalin solution for histology. The placenta is then weighed and a third photograph is taken before safe discarding.

Maternal and newborn stool samples are being also collected to assess microbiota around the time of delivery and when newborn feeding is established, respectively. A single sample of paternal taken either during one of the antenatal or postnatal visits) and two of newborn saliva (are taken at 42–60 days postpartum from babies whose cord blood could not be obtained at the time of birth) are collected using an Oragene DNA collection kit for DNA extraction. All biological samples are stored at –80°C.

**Outcomes.** The main adverse pregnancy outcomes being evaluated in the AMANHI bio–repository study are (pre–) eclampsia, intrauterine growth restriction, preterm birth and stillbirths.

**Sample size considerations.** In this study, 3000 pregnant women will be recruited from the three participating sites (1000 per site) over a period of one year. Sample size and power considerations were based on the 3% prevalence of pre–eclampsia (the rarest outcome) in the population. The 3000 women from the three sites is only sufficient to detect a 1% (absolute) change in the prevalence of pre–eclampsia with 90% power and at 5% significance level if analyses adopt a case–control design with a 1:3 ratio of cases to controls.

**Confidentiality.** All participants are provided with unique study IDs with which they and their families are identified in the study. All data collected from participants are being kept confidential; hard copies of study–related forms are stored in locked cabinets and soft copies are securely stored on dedicated, password–protected servers. These are only accessible to the principal investigators and approved co–investigators. After completion of the study, any sample to be used for analyses will be de–linked from participant’s identity and only alphanumeric identification numbers will be used.

**Safety issues in sample collection and processing**. All the personnel involved in sample collection and processing are well trained by the sites. AMANHI–specific standard operating procedures (SOP) have been developed and all phlebotomists and laboratory scientist involved have been trained in study participant care, sample collection, sample processing, bio–safety in sample handling and safe disposal of instruments and materials (**Online Supplementary Document(Online Supplementary Document)**).

### Bio–specimen storage and security

**Administration.** The principal investigators of the three sites are responsible for the implementation and management of the study. These principal investigators will be responsible for maintenance of optimal quality of the biological samples and will lead all analyses to be conducted as part of the study. They have, in consultation with governmental agencies and ministries of health, academia and key stakeholders, constituted a governance council to take over the management of the biobanks after the main AMANHI analyses are completed. These governing councils will meet at agreed times and at various frequencies to receive, review and approve protocols for studies that require the use of the AMANHI biological samples in the biobank.

**Temperature maintenance.** Maintenance of optimal storage temperature of all biological samples is a key issue in the biobanks and is a core component of the common protocols being used across sites. There are minor site–specific adaptations; for instance, in Karachi and Pemba, to minimise the travel distance between the families and the biobank, field laboratories have been set up and samples are transited through shuttle freezers (also maintained at between –76°C to –86°C) to the Biobank. Temperature logs are maintained for shuttle freezers during transport and main freezers at the biobank.

**Power supply and alert systems.** Availability of reliable power supply is cited as one of the reasons why biobanking may not be feasible in LMICs and so premium was placed on this [25]. All sites have therefore procured additional power back–up generators as well as high capacity uninterruptible power supply (UPS) systems to protect sensitive equipment from power trips, surges and fluctuations. For instance, the Pemba site has installed a 18KV solar power system with 2 days autonomy as one of the power backup system other than national grid and generators. The biobanks have also been fitted with security alarm systems that immediately report through text messages and emails (to the principal investigators and appropriate technical persons for immediate redress) any temperature fluctuations and power trips irrespective of whether the back–up systems are activated. All these have been piloted and are being closely monitored.

**AMANHI Biobanking software.** A special Windows–based software has been developed for the capture of the AMANHI biospecimen collection. This software which was developed by the Pemba team is aimed first at reducing transcription errors in the data capture and to align the sample collection process with the common protocol. It has in–built range and consistency checks and will only accept data appropriate for the field in which it is being entered. All samples and study materials are provided labels with encrypted 2D digital signature codes that are read by a 2D scanner into the software. The software has undergone several rounds of testing before deployment to all the sites for use. Training has been provided to all the sites on the installation and the use of the software for data capture and real–time troubleshooting is being done for all sites because individual sites do not have the facility to edit the database. In case of any errors they have to inform the WHO coordinator for the study (Leader, MCA/MRD, Newborn Health Research) who has the password. A specified software expert does the required changes if it is essential and then the database is again locked.

**Coordination, monitoring and quality assurance.** The Maternal, Newborn, Child and Adolescent Health department (MCA) of the World Health Organization (WHO) is centrally coordinating the study. This involves technical input into the implementation as well as raising contracts with the constituent sites for the implementation. The WHO team has also procured the services of various experts from the United States of America, the University of Edinburgh in Scotland and Croatian National Biobank [31] to make specific input into various aspects of the study and to maintain the quality of implementation. Weekly (initially, but now fortnightly) teleconferences are held between team members from all the sites, the WHO team and the experts to discuss implementation challenges and to make decisions on the progress and strategies for the implementation. In preparation for these, sites present a progress report to the WHO which is discussed during the teleconferences. The experts are also commissioned at quarterly intervals to make visits to the sites to monitor the progress and quality of the implementation. After each visit, a detailed feedback is provided to the site and a report is also submitted to the MCA. In between site visits, the experts conduct videoconferences with the sites to monitor implementation. Other monitoring processes have been described in the other AMANHI publications [26,27].

**Ethical considerations.** The biomarker study has received ethical approval from the local and institutional ethics committees of all the three sites: ICDDR,B and John Hopkins University for Bangladesh, Aga Khan University for Pakistan and ZAMREC and John Hopkins University for Tanzania. The protocols were also approved by the WHO Ethics Review Committee and continuing approvals are obtained each new year. There will be no direct benefits of the study to the participants. They will be compensated for the time contribution to the sample collection at the health facilities.

**Plan for analyses.** Analyses of the epidemiological data will be carried out to characterize the women in the study and to link the various reported and measured exposures to the main outcomes. These will include principal components analyses (PCA) at each site to generate asset indices from the inventory of assets collected. These indices will be ranked and divided into quintiles and individual women will be assigned the wealth quintiles for their household. Associations will be explored using simple cross tabulations and tested with either a χ^2^ or Fisher exact tests for their significance. Where applicable, models with robust standard errors will be fitted to explore relationships. Multivariable regression models will be fitted in the risk factors analyses and likelihood ratio tests used to assess statistical significance.

**STEP 1: Testing existing hypotheses**. Simple, highly focused analyses to identify current hypothesized biomarkers associated with the risk of the main outcomes will be conducted using small aliquots of the biological samples. The analyses will seek to:

1. replicate the role of previously identified genetic variants which have been identified as important determinants of these outcomes in high income countries (HICs) through genome–wide association and candidate gene approaches and explore a panel of candidate genetic variants;

2. evaluate the validity of the most commonly proposed existing biochemical markers (in serum, plasma or urine) of these outcomes.

**STEP 2: Exploratory “hypotheses free” research using the biobanks**. Using high throughput “hypothesis free” approaches will be done to advance the science of biomarker–disease pathway discovery. This will include analyses of data from whole–genome arrays, high throughput data on many “–omics” traits, relevant maternal and newborn health outcomes and disease phenotypes. These will be “data driven” analyses in which novel high throughput technologies will be employed to yield high dimensional genomic, proteomic, lipidomic, glycomic and epigenetic data on large sample sizes to discover and identify entirely new associations and biomarkers. These analyses will require substantial financial investment and will be conducted through collaborations with other experts within countries and around the globe.

**Plan for dissemination of findings.** The results of the AMANHI biomarker study will be disseminated among the public health and maternal and newborn health community of researchers, policy–makers and programme managers. Channels for dissemination will include peer–reviewed journals, print and electronic media and through oral and poster presentations at appropriate fora. In each participating country, there will be extensive briefing on their country–specific and overall study results with interpretation of the potential implications for health programmes to the country.

## DISCUSSION

We present here the protocols used for the harmonised implementation of, to the best of our knowledge, the first population–based harmonized multi–country bio–repository study to be set up in developing country settings. With data on phenotypic and epidemiological characteristics and most importantly epigenetic and biochemical information, this study may have the best characterized cohort of pregnant women and their newborns in the entire developing world; certainly so in south Asia and sub–Saharan Africa. Successful implementation of the study affords the opportunity to explore hypothesized risk factors for adverse pregnancy outcomes and also allow further explorations into biomarker–disease pathway along several dimensions in LMICs.

Hitherto, biobanking has largely been the preserve of developed country settings [19,20]. There is however a systematic challenge in translating research results from such settings to developing country settings. Also whilst non–communicable diseases tend to be the main agenda for biobanks in HICs and hence a lot of emphasis is on adult health outcomes, infectious diseases as well as preventable maternal, newborn and child deaths will require much greater focus in developing country settings [20]. There are also systematic differences in exposures to various risk factors due to differences in culture, workplace environment, lifestyle and access to health care. In contrast to HICs, vital registration systems are non–existent in LMICs, health systems are weak and majority of the population do not have access to health services and hence a facility–based bio–repository will have significant biases and may not be representative. It is believed that when biobanks are sited in LMICs, they will likely address specific needs and equity considerations [20,32,33].

A review by McKinnon et al [7] on birth cohort studies in South East Asia and Eastern Mediterranean found only few studies that saved biological samples. Most of these studies had small sample sizes and were of relatively low quality. Only one of the studies had DNA samples stored for later analyses and none took genetic material from family members. Other logistical challenges such as availability of reliable power supply, the ability to assemble sufficient human resource with the capability of setting up such biobanks in LMICs are often thought of as reasons why such biobanks will not be feasible in LMICs [25] which carry over 95% of the world’s burden of morbidity and mortality around childbirth [34].

The AMANHI study proves the principle that such biobanks are feasible to set up in developing country settings. The AMANHI sites are collecting population level data that is very representative of rural populations is the study settings. The inclusion of genetic material and data from family members that can be used directly or “as proxies of exposure” for the identification of “parent–to–origin effects and de novo mutations” is quite novel in these developing country settings. It will therefore contribute to knowledge in the testing of existing hypothesis around risk factors for adverse pregnancy and birth outcomes in these LMICs of sub–Saharan Africa and south Asia who carry the highest burden of these. It will establish a platform for further exploration of new hypotheses and technologies and build local capacity in these low–resource settings for high quality research and ultra–high throughput analyses. Its analytical approach including “hypothesis–free” analyses has been described as one that is potentially free of human biases and may create opportunities for breakthrough discoveries in the biomarker–disease pathways [19]. Its implementation provides a model for adaptation in conducting high impact research in similar settings.

The key desirable attributes of such studies are clear aims and objectives, cultural and social acceptability to both participants and staff involved and low attrition rates. AMANHI has all these attributes. The harmonized implementation including the use of uniform protocols, centralised procurement of equipment, materials and reagents and the strict and rigorous quality control measures will allow for comparability of data across all sites which will allow for pooling and consequent use in the analyses of rare outcomes. A significant limitation of the AMANHI bio–repository study is its inability to inform on biological mechanisms underlying childhood linear growth and neurodevelopment. With the best characterized population based cohort in these LMICs, following up children into the 2nd and 3rd years after birth would have provided opportunities to examine epigenetic factors in pregnancy or early childhood that predict stunting and impaired neurodevelopment, the origins and risk factors of susceptibility to infectious agents and non–communicable diseases.

Establishing these biobanks is only the first step and will be of little value of it is not utilized to address current global challenges. Strategic investment to maximise the utility and gains from this infrastructure is an ethical call. This investment will allow for the use of existing and newly developed ultra–high throughput technologies and develop local capacity to participate and use such technologies to address global health challenges.
