# Supplementary material for: Understanding biological mechanisms underlying adverse birth outcomes in developing countries: protocol for a prospective cohort (AMANHI bio–banking) study
Source: J Glob Health. 2017 Nov 1;7(2):021202. doi: 10.7189/jogh.07.021202 (PMC5665675; doi:10.7189/jogh.07.021202)
Supplement: Online Supplementary Document [file jogh-07-021202-s001.pdf]

Online Supplementary Document

AMANHI Bio–banking study et al. Understanding biological mechanisms underlying adverse birth outcomes in developing countries: protocol for a prospective cohort (AMANHI bio–banking) study

J Glob Health 2017;7:021202

Sample handling

Blood samples are collected into pre-labelled specimen containers assembled as study kits. Approximately 15ml of maternal venous blood is collected from each study participant by trained phlebotomists, under aseptic conditions, at the scheduled visits. Similarly, 15ml of cord blood is collected after delivery. Blood is centrifuged at 4°C at 2000g rotation for 15 minutes to obtain samples of the serum, plasma and the buffy coat. Aliquots of whole blood samples are placed on a Whatman card and dried and additional aliquots for haemoglobin A1c (HBA1c) assay. If cord blood is clotted, fetal veins are dissected and clotted blood samples are harvested. Processed samples are stored in -80°C freezers.

About 50ml of maternal urine samples are collected into a sterile 90ml transport container. Aliquots are taken into plain S-monovette tube with mineral oil and five other empty tubes. A sub-sample of the urine is centrifuged at 4°C at 2000g rotation for 15 minutes. RNALater is mixed with the sediment and aliquots taken into pre-labelled colour-coded tubes.

Placenta samples are harvested and processed within 30 minutes of delivery. The placenta is placed between two perpendicularly placed rulers on a dissecting board to help estimate the surface area and photographed (fetal and maternal surfaces). Full thickness tissues samples are harvested in four areas, three of which have a thin layer of maternal tissues sliced off the surface. These samples are stored in RNALater, alcohol, or are flash frozen. A sample is also harvested and stored in formalin solution for histological analyses. A sample of the placental membrane and cross-sections of the umbilical cord are also taken. The placenta is then weighed and a third photograph of the placenta is taken before discarding in a bio-hazard waste container.

Maternal and newborn stool samples are being collected in order to assess their microbiota around the time of delivery and when newborn feeding is established, respectively. Stool samples are stored at between 2°C to 8°C during transport to processing laboratories where a 100mg of the sample is taken and placed into a pre-labelled sterile faeces tube and capped for storage in the -80°C freezer.

A single sample of paternal saliva for all enrolled women and newborns and two samples of newborn saliva are collected using an Oragene DNA collection kit and stored in a -80°C freezer. The paternal sample is taken either during one of the antenatal or postnatal visits

and the newborn saliva samples are taken at 42-60 days postpartum from babies whose cord blood could not be obtained at the time of birth. DNA will be extracted from these samples.
